# Comparative Evaluation of Voids Present in Conventional and Capsulated Glass Ionomer Cements Using Two Different Conditioners: An *In Vitro* Study

**DOI:** 10.1155/2014/935240

**Published:** 2014-12-03

**Authors:** Mamta Kaushik, Roshni Sharma, Pallavi Reddy, Pallavi Pathak, Pooja Udameshi, Narmatha Vallakuruchi Jayabal

**Affiliations:** Department of Conservative Dentistry and Endodontics, Army College of Dental Sciences, Chennapur, CRPF Road, Jai Jawahar Nagar Post, Secunderabad, Andhra Pradesh 500087, India

## Abstract

This *in vitro* study evaluated the presence of voids in powder-liquid and capsulated glass ionomer cement. 40 cavities were prepared on root surfaces of maxillary incisors and divided into four groups. Cavities were conditioned with glass ionomer cement liquid (GC Corporation, Tokyo, Japan) in Groups 1 and 3 and with dentin conditioner (GC Corporation, Tokyo, Japan) in Groups 2 and 4. Conventional powder-liquid glass ionomer cement (GC Fuji II, GC Corporation, Tokyo, Japan) was used as a restorative material in Groups 1 and 2. Capsulated glass ionomer cement (GC Fuji II, GC Corporation, Tokyo, Japan) was used in Groups 3 and 4. Samples were sectioned and viewed under stereomicroscope to check for the presence of voids within the cement and at the cement-tooth junction. Data was analyzed using one-way ANOVA and Tukey's post hoc tests. Group 4 showed statistically significant results (*P* < 0.05) when compared to Groups 1 and 2 for voids within the cement. However, for voids at the margins, the results were statistically insignificant.

## 1. Introduction

Glass ionomer cement is a popular direct restorative material in dental practice. It has certain advantages like physical/chemical bonding to tooth structure, anticariogenic property due to fluoride release, esthetics, and low coefficient of thermal expansion [1]. On the other hand, poor mechanical properties, such as low fracture strength, toughness, and wear resistance, limit its use as a filling material to low stress-bearing areas [2, 3]. Glass ionomer is indicated for restoration of primary teeth, core build-up, root surface lesions, and restorations of Classes III and V and some Class I cavities.

Surface treatment of the cavity by a conditioning agent is advocated as a prerequisite to GIC restoration. Conditioning removes the smear layer and the acid partially demineralizes and penetrates the superficial dentin surface up to 1 *μ*m [4]. Two conditioning agents, that is, glass ionomer liquid containing 40% polyacrylic acid as a copolymer with itaconic, maleic, or tricarboxylic acid and tartaric acid and distilled water and dentin conditioner containing 10% polyacrylic acid, are generally used for this purpose.

Predosed capsules were introduced to overcome the problems associated with manual mixing due to improper ratio and consistency. Several workers have reported that mechanical properties of encapsulated materials were equivalent or inferior to those of the hand-mixed materials [5, 6].

The aim of the present study was to compare the presence of voids in conventional and capsulated GIC using two different conditioners.

## 2. Materials and Methods

### 2.1. Specimen Preparation

For this study 40 freshly extracted maxillary incisors were collected from the Department of Oral and Maxillofacial Surgery. Criteria for tooth selection included: a single root, no visible root caries, fractures, or cracks, and absence of root resorption.

The teeth were cleaned of gross debris, scaled with ultrasonic instruments, washed with distilled water and sterilized in an autoclave.

**Figure 1:**
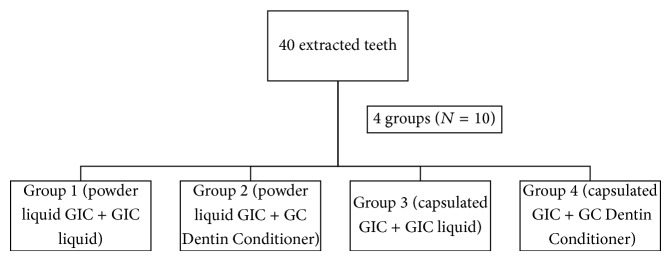
Distribution of samples.

Samples were randomly divided into 4 groups, with size 10 each (Figure 1).

**Figure 2:**
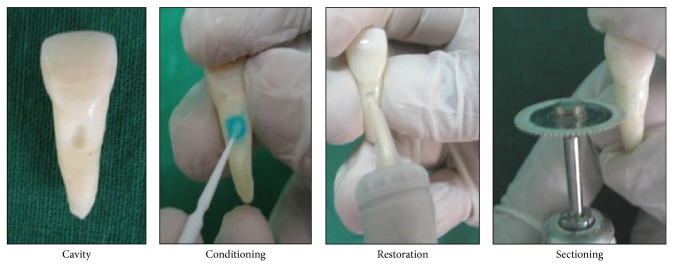
Procedure.

Rectangular box shaped cavities of the dimensions 2 × 2 × 3 mm were prepared longitudinally on the buccal root surface of each tooth. Initial punch cut of 1.5 mm was prepared with a 3 mm diameter round diamond, mounted on a high speed hand piece. The cavities were then made rectangular with a flat end straight fissure bur no 56 (Figure 2).

### 2.2. Restorative Procedure

*Group 1*. Cavities were conditioned with GIC type II universal restorative liquid. It was applied on the dentin surface with an applicator tip and left for 20 seconds after which it was washed with water. Cavities were air-dried but not desiccated and restored with type II universal restorative powder and liquid. After mixing the cement manually in a ratio of 1 scoop powder , 1 drop liquid, it was carried to the cavity and condensed with a plastic filling instrument.

*Group 2*. Cavities were treated with GC Dentin Conditioner for 20 seconds, washed with water, and air-dried but not desiccated. They were restored in the same manner as described in Group 1.

*Group 3*. Conditioning of the cavities was done as in Group 1 followed by restoration with GC Fuji II Capsules. Before activating, the capsule was tapped on a hard surface to loosen the powder and the plunger was pushed until flushed with the body of the capsule for activation. It was then placed into an amalgamator (Dental Amalgam Mixer SYG—200) and mixed for 2 seconds (4,300 RPM). The nozzle of the capsule was inserted into the cavity to contact the axial wall and withdrawn filling the cavity from inside out, without using any hand condensation (as per manufacturer's instructions) following which excess cement was removed.

*Group 4*. Conditioning was done with GC Dentin Conditioner as described in Group 2 followed by restoration with GC Fuji II Capsules as in Group 3 (Table 1).

All the samples were coated with a protective layer of petroleum jelly and stored in 100% humidity at room temperature for 24 h. This was followed by sectioning the samples at the centre of the cement in a mesiodistal direction with the help of a diamond saw (Confident Mighty Lab Digi C-108 A).

The sections were then examined at 40x magnification under a stereomicroscope (Leica Microsystems, done at RCMA, DRDO Labs, Hyderabad, India).

**Figure 3:**
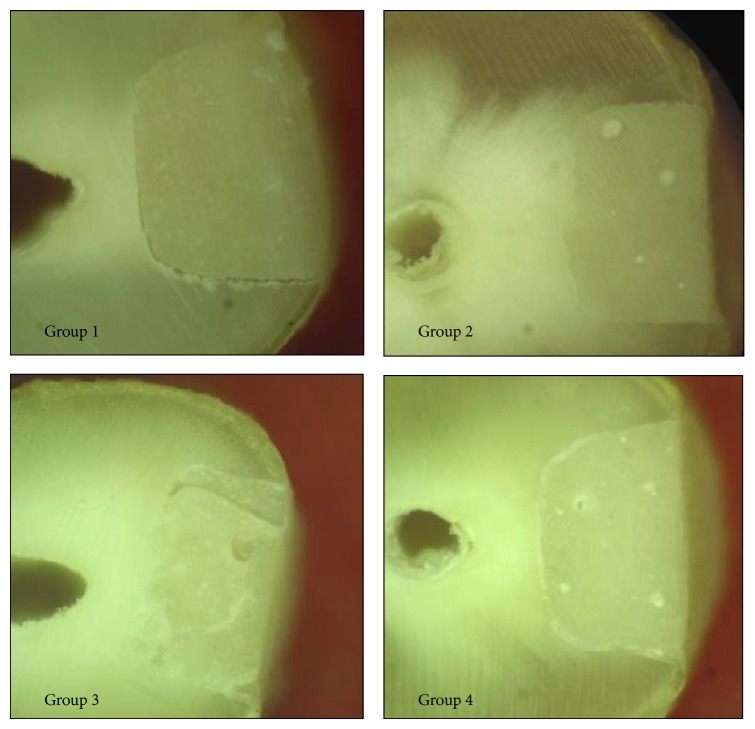
Results.

Stereomicroscopic images were examined and evaluated for voids within the cement and at the cement-tooth junction as shown in Figure 3.

The maximum width of the void at the margins was measured in *μ*m. The number of distinct, round voids within the cement was counted independently by 3 different observers for each sample and mean values were obtained.

## 3. Results

Results were analyzed using one-way ANOVA test with SPSS 11.5 software, to obtain the sum of squares and significance levels between the groups. A *P* value of < 0.05 was considered to be significant. The groups evaluated for voids within the cement yielded a significant difference with *P* = 0.002. This was confirmed using Tukey's post hoc test. Group 4 showed statistically significant results (*P* < 0.05) when compared to Groups 1 and 2 for voids within the cement.

For voids at the margins, the difference was insignificant with *P* = 0.996 (Tables 2, 3, and 4).

## 4. Discussion

Glass ionomer cement is a direct tooth colored restorative material. Among its many indications, it is the material of choice for root surface lesions. The ideal prerequisites for a root surface restorative include good marginal seal, low microleakage, low solubility, radiopacity, anticariogenicity, and esthetics. As there is no occlusal load, the physical properties of the material may not be very significant. The present study analyzed the void pattern within the root surface restoration.

Capsulated GIC claims easy handling and standardized and high *P*/*L* ratio and homogenous consistency compared to the powder-liquid counterpart. Hence, capsulated GIC was compared with powder-liquid GIC.

Following cavity preparation, a smear layer is formed on the surface of dentin. Studies have shown that this layer can impede the intimate contact of glass ionomer to dentin and consequently compromise the chemical and/or micromechanical interaction [7, 8]. This was confirmed by a study done by Mauro et al. [1], in which the lowest bond strength values were observed when dentin did not receive any pretreatment. The bond strength values improve from 1–3 MPa to 11 MPa by conditioning [9, 10]. Pretreatment with a diluted polyacrylic acid conditioner has the ability to remove smear layer and partially demineralize the dentin [8, 11]. The hydroxyapatite left around exposed collagen fibrils becomes accessible for chemical interaction [12–14] with the carboxylic groups from GIC [7, 15–17]. This is expected to ensure a good bond between GIC and dentin, free of voids.

GC Fuji II liquid is commonly used to condition the dentin surface prior to restoration as it contains polyacrylic acid. The manufacturers recommend GC Dentin Conditioner (10% polyacrylic acid) which is a mild acid composed of large molecules thereby being more biocompatible when used in tooth repair [1]. It has the advantage of low viscosity as compared to Fuji II liquid. This might allow for better wetting of the dentinal surface and better conditioning of the cavity. The blue tint allows application control. However, there is a lack of evidence depicting the correlation between the conditioning agents used with voids at margins.

Voids within the cement act as a source of stress concentration, making the cement more brittle [18]. The homogeneity and strength of the material are compromised. Therefore, a homogeneous mix is preferred. Manual mixing is speculated to produce more voids and less homogeneity due to air entrapment as compared to automixing. This has been supported by Jorgensen et al. who have found 3.5% porosity in hand-mixed cements [19]. Mitchell et al. have found lower fracture toughness for hand-mixed luting GIC [20]. The possible reason could be based on the findings that thin layers (40 *μ*m) of hand-mixed conventional glass ionomer cements contain greater numbers of large diameter defects (0.05–0.6 mm) than the comparable capsulated cement [21].

According to Jones et al., reduced cement viscosity resulted in increased porosity [22]. Depending on GIC viscosity, Nomoto et al. found a 10% decrease in strength at 0.2% porosity in a restorative GIC [5].

In contrast, Nomoto and McCabe found more bubbles during mechanical mixing [23]. Aws has proved that the encapsulated glass ionomer cement has more pores of diameter 1–10 *µ*m than hand-mixed cement [6]. This is in agreement with the current study where more voids within the cement have been found in Groups 3 and 4 where GIC capsules were used.

In the current study, better results with hand-mixed GIC can also be explained on the basis that hand condensation may have resulted in more compact cement. Rapid mixing of the mechanical mixing process may cause air inclusion, whereas slower mixing of hand-mixing procedure in which the material is spatulated may avoid these inclusions and collapse some air bubbles. Also, the mechanically mixed glass ionomer was inserted passively into the cavity as per manufacturer's instructions in contrast to the hand-mixed GIC which was condensed, probably lessening the number of voids. Further studies are required to correlate the presence of marginal voids with increased marginal leakage and decreased bond strength and the impact of the number of voids within the cement to the physical properties of the restoration.

## 5. Conclusion

Within the limitations of the current study, hand-mixed powder-liquid GIC had less number of voids as compared to its capsulated counterpart. Dentin conditioner and GIC liquid were equally good at preventing void formation at cement-tooth interface.

## Figures and Tables

**Table 1:** Materials, composition, and manufacture.

Materials employed	Composition	Manufacturer
GC Fuji II Universal Restorative	Powder: 95% fluoroaluminosilicate glass (amorphous) 5% polyacrylic acid	G.C. Corporation, Tokyo, Japan
	Liquid: 50% distilled water 40% polyacrylic acid	

GC Fuji II Capsule	Powder/liquid ratio (g/g) 0.30/0.11	G.C. Corporation, Tokyo, Japan

GC Fuji II Universal Restorative Liquid	50% distilled water 40% polyacrylic acid	G.C. Corporation, Tokyo, Japan

GC Dentin Conditioner	10% polyacrylic acid	G.C. Corporation, Tokyo, Japan

**Table 2:** Mean values for voids within the cement.

Groups	Mean	Standard deviation	Decision^*^
Group 1	7.08	2.87	a
Group 2	7.35	2.74	a
Group 3	10.39	5.16	b
Group 4	13.23	3.83	b

^*^Different letters indicate statistically significant differences between groups (*P* < 0.05).

**Table 3:** Mean values for voids at the margins (*μ*m).

Groups	Mean	Standard deviation	Decision^*^
Group 1	12	26.99	a
Group 2	16	50.59	a
Group 3	14	44.27	a
Group 4	12	37.97	a

^*^Different letters indicate statistically significant differences between groups (*P* < 0.05).

**Table 4:** Results of Tukey's post hoc test.

Dependent variable	Group	Group	Mean difference	Significance
Voids within cement	4	3	2.84	0.349
		2	5.88	0.007^*^
		1	6.15	0.005^*^
	3	2	3.04	0.291
		1	3.31	0.223
	2	1	0.27	0.999

Values marked with ^*^indicate statistically significant differences.
